# Dr. H. P. Sharp

**Published:** 1908-03

**Authors:** 


Dr. H. P. Sharp, of Arcade, N. Y., died at his home in that village January 29, 1908, aged fifty-five years. Dr. Sharp had been engaged in the practice of his profession at Arcade for about twenty years, removing thence from Varysburg.

